# Prevalence and Mechanisms of Dynamic Chemical Defenses in Tropical Sponges

**DOI:** 10.1371/journal.pone.0132236

**Published:** 2015-07-08

**Authors:** Sven Rohde, Samuel Nietzer, Peter J. Schupp

**Affiliations:** Institute for Chemistry and Biology of the Marine Environment (ICBM), Carl-von-Ossietzky University Oldenburg, Wilhelmshaven, Germany; Victoria University Wellington, NEW ZEALAND

## Abstract

Sponges and other sessile invertebrates are lacking behavioural escape or defense mechanisms and rely therefore on morphological or chemical defenses. Studies from terrestrial systems and marine algae demonstrated facultative defenses like induction and activation to be common, suggesting that sessile marine organisms also evolved mechanisms to increase the efficiency of their chemical defense. However, inducible defenses in sponges have not been investigated so far and studies on activated defenses are rare. We investigated whether tropical sponge species induce defenses in response to artificial predation and whether wounding triggers defense activation. Additionally, we tested if these mechanisms are also used to boost antimicrobial activity to avoid bacterial infection. Laboratory experiments with eight pacific sponge species showed that 87% of the tested species were chemically defended. Two species, *Stylissa massa* and *Melophlus sarasinorum*, induced defenses in response to simulated predation, which is the first demonstration of induced antipredatory defenses in marine sponges. One species, *M*. *sarasinorum*, also showed activated defense in response to wounding. Interestingly, 50% of the tested sponge species demonstrated induced antimicrobial defense. Simulated predation increased the antimicrobial defenses in *Aplysinella* sp., *Cacospongia* sp., *M*. *sarasinorum*, and *S*. *massa*. Our results suggest that wounding selects for induced antimicrobial defenses to protect sponges from pathogens that could otherwise invade the sponge tissue via feeding scars.

## Introduction

Sponges and other sessile invertebrates are lacking behavioural escape or defense mechanisms and rely therefore on morphological or chemical defenses. Already in the 1950s it was discovered that sponges yield a wide array of biologically active secondary metabolites [1]. Since then more than 5300 chemical compounds have been described in sponges [2]. Marine natural products research has been driven mainly by pharmacological screening programs, which aim at the discovery of new chemical structures with pharmacological activity, neglecting the ecological functions of these compounds. Studies in the field of marine chemical ecology focus on chemically mediated ecological interactions and revealed a pronounced bioactivity of many sponge compounds, e.g. defense against predation, fouling or pathogen attack [3–6].

While past studies in chemical ecology have treated sponges mainly as whole organisms neglecting their ability to house high numbers of microbial symbionts (up to 40% of the sponge biomass [7]), recent studies are more and more trying to evaluate the role of microbial symbionts in the production of biologically active secondary metabolites. So far, only a few studies have identified the actual producers of secondary metabolites of interest, implicating either the sponge itself [8, 9], or the associated bacteria [10–12]. Regardless of the actual producer of bioactive secondary metabolites, the sponge itself, or the associated microorganisms, sponge holobionts (sponge plus the associated microorganisms) often defend themselves against predation by producing feeding-deterrent compounds (e.g. [3, 13, 14]).

Terrestrial plant defense theories have been used as the framework of choice for investigations of defense in other systems (e.g. [15, 16, 17]). This framework is of particular relevance to marine sessile invertebrates like sponges, since they are comparable to plants in their ecological traits as being sessile, liberate dispersive propagules, and produce a diverse array of secondary metabolites. In recent years, several marine studies supported findings on variable defense strategies originally described from terrestrial systems [18, 19]. These studies indicate that in addition to the simple production and storage of “chemical weapons” in their tissue, algae, sponges, and other sessile marine invertebrates evolved mechanisms to increase the efficiency of their chemical defense. These mechanisms will likely save metabolic energy invested in their defenses [20, 21] and protect them from cell damage caused by their own bioactive defense compounds [22]. These mechanisms could also represent evolutionary adaptations in response to different biological threats. Defense types can be divided in three strategic categories, each being optimal for a specific threat: constitutive, activated and induced defense.

1. *Constitutive defense*, should be favoured when the sessile organisms (e.g. sponges) live under constant predation pressure and/or predators are dominated by highly motile macropredators (e.g. fish). Given the example of sponges, macropredators are capable to consume large amounts of sponge biomass in short time periods and are fast moving animals that do not spend much time at one prey individual. An induction of chemical defensive compounds could include high rates of biomass loss during the induction period.
2. *Activated defense*, can be regarded as specialised form of a constitutive defense. Precursors of bioactive compounds are produced, stored and following wounding, converted to potent defensive products [23]. The conversion is fast (e.g. seconds) and often enzyme catalysed. By converting inactive or less active precursors to highly active defense metabolites only upon tissue damage and locally restricted to the wounded tissue, the risk of autotoxicity by the conversion products can be alleviated.
3. *Induced defense*, is defined as the ‘de novo’ production of defensive compounds activated by predator attack. Induction of defense includes that defensive compounds are only produced on demand, thereby saving resources and increasing the prey’s chemical variability [18, 21, 24, 25]. A disadvantage of induced defenses is however, that the induction process requires several days to weeks during which the sponge is undefended and could suffer from high biomass loss. Induced defense is therefore regarded as favourable under variable grazing conditions and/or the dominance of mesopredators like small crustaceans or gastropods that feed at slow rates and biomass loss within the induction period is tolerable [18, 24].

Defensive strategies should have evolved in response to the dominant regional predation patterns. Consequently, in regions with constant predation pressure and a dominance of macropredators like fish (e.g. coral reefs), sponges should display constitutive or activated defenses [26]. In the marine environment, research on activated chemical defenses is just beginning. So far, most activated defenses have been discovered in algae, while very few examples of this strategy have been described in sessile marine invertebrates [27]. However, having reviewed defensive strategies in sponges, Thoms & Schupp (2007) hypothesized that activated defenses may have been overlooked in the past due to traditional and restricted methodological approaches by marine chemical ecologists and that “various factors could possibly have led to underreporting of activated chemical defenses in the past, bolstering constitutive defense as the status quo in sponge chemical defense.”

In contrast, in regions where sponges experience variable predation pressure and mesopredators like crustaceans or sea urchins are the major consumers (e.g. temperate rocky reefs), inducible defenses are discussed as favored defensive strategy, since the slow grazing rates of the regional predators allows to tolerate the low biomass loss within the induction period [18, 24, 26].

Tropical and temperate benthic systems differ with regard to dominant consumption regimes. While in tropical regions fishes are the dominant predators on reefs, temperate systems are predominantly characterised by a mesopredator community of crustaceans, sea urchins or gastropods [26]. In Guam, which is surrounded by fringing coral reefs, the major sponge consumers are fish. Due to the tropical conditions, where seasonal changes in water temperature and light intensities are almost lacking, the variability in grazer density is low. These prerequisites should favour activated or constitutive defense.

Activated defense mechanisms have been widely recognized in terrestrial plants [21, 28] and to a lesser extent in marine algae [23, 29, 30]. However, examples of activated defense in marine sessile invertebrates have been very limited, with two examples from the Verongid sponges *Aplysina* spp. [31, 32] and *Aplysinella* sp. [33] and the hydroid species *Tridentata marginata* [34]. Despite these examples, the existence of activated defense in sponges has been discussed very controversially (see [31, 35]). Similar patterns have been described with induced defenses. Defense induction is widely known in terrestrial plants [21] and marine macroalgae [18, 19], but studies on on marine invertebrates are scarce. While the sponge *Chondrilla nucula*did not induce defense [36], the sponge *Agelas conifera* increased concentrations of feeding deterrent compounds after simulated predation [37].

Antimicrobial activity is a very common feature of sponge extracts [38, 39]. However, induced antimicrobial activity has not been found in sponges, and activated antimicrobial activity was demonstrated in only one sponge species [31].

From terrestrial studies we know that induced defenses can impact both consumers and pathogens [40], and are in fact even caused by microbial pathogens [41]. This has also been shown in a freshwater macrophyte, where induced antifeeding defense was accompanied by an induction of antimicrobial defense [42].

The effects of predator feeding versus invasion by microbial pathogens via grazing scars on the induction process is unclear [42]. Induced responses could be due to feeding or to pathogens that infect the prey via wounds made by predators [41, 43]. Thus, predation may not only harm prey organisms directly, but could also increase the risk of pathogen infection. Microbial attacks are especially likely in aquatic systems, where microbial densities are much higher (10^4^-10^7^ cells ml^-1^) than atmospheric densities [44].

This study tried to evaluate chemical defense mechanisms in eight different sponge species. Experiments were run under controlled conditions to exclude multiple factors in the field that increase chemical variability [45]. Specifically, we tested for presence of constitutive defenses, induced and activated defenses by examining the feeding deterrence and antimicrobial activity of sponge extracts.

The results of this study will provide extensive insight in the prevalence of induced and activated chemical defense mechanisms of sponges and identify whether induced or activated responses also suppress co-occurring microbes that might function as pathogens.

## Materials and Methods

### Organisms and defense experiments

We chose eight sponge species that were abundant members of the sponge (Demospongia) community on Guam’s reefs and represent a wide taxonomic range: *Aplysinella* sp. (Aplysinellidae), *Dysidea granulosa* (Dysideidae), *Cacospongia* sp., *Hyrtios altus* (Thorectidae), *Iotrochota* sp. (Iotrochotidae), *Stylissa massa* (Dictyonellidae), *Neopetrosia carbonaria* (Petrosiidae) *and Melophlus sarasinorum* (Geodiidae). All sponge specimens were collected around Guam (13.4° N, 144.6° E) at depths between 2–15 m. 19 specimens of each species were cut at their base from the substratum, put into individual 4-L plastic bags *in situ*, then deposited into a cooler filled with seawater for transportation to the University of Guam Marine Laboratory (UOGML). Collections were not conducted in Marine Protected Areas and only non-CITES species were collected. Therefore, no collection permit was required. At the UOGML the sponges were placed on U-shaped concrete blocks in a 1500 L outdoor flow-through tank to heal and adjust to the experimental conditions for one week. During this time the sponge bases healed and reattached onto the blocks.

Two experiments were carried out with the sponge species. The first experiment was designed to test for defense induction, the second to test for defense activation. After both experiments, feeding assays with two spongivorous species were conducted.

Defense induction experiments were run in outdoor flow through tanks at the UOGML. Ambient seawater was individually supplied (25 L•h-1) to twelve 60 L plastic aquaria, each containing one sponge specimen. In preliminary experiments, puffer fish and sea urchins were used as predators to induce defenses. However, no regular feeding was observed in the aquaria. Consequently, we used clipping as artificial predation treatment in the experiments. To test for induced defenses six sponges were artificially grazed by removing small tissue pieces from the sponges with forceps in order to mimic fish bites (induced treatment). Five pieces (10–100 μl volume) per day were taken randomly from different spots on the sponge surface over the course of two weeks. The other six sponges served as controls and were left intact (non-induced control). After 14 days of treatment, the volume of all sponges was measured by seawater displacement (sponges were put in a seawater-filled bowl and the displaced volume of water was measured in a graduated cylinder). Then sponges were frozen, freeze-dried and extracted. Defense activation was tested by dividing seven specimens of each species into two halves. One half was put straight into a -80°C freezer (non-activated control). The other half was ground in 10 ml seawater for 2 min to destroy cell compartmentalization and allow enzymatic conversion of compounds and then frozen along with the seawater at -80°C (activated treatment [33]), before being freeze-dried and extracted as described below.

### Extraction and feeding assays

Freeze-dried sponges were ground to a fine powder in a blender. The materials of each group (activated, non-activated, induced, and non-induced) were pooled and extracted exhaustively in methanol / ethyl acetate (1:1). The extracts were filtered and the solvent removed under vacuum by rotary evaporation. The extracts were then incorporated at natural volumetric concentration in an artificial diet, following a modified method by Hay et al. [46] and Schupp et al. [47]. Commercial catfish food (Rangen Extruded 350, 2 g, ground to a powder), Agar (0.36 g) and distilled water (18 ml) were mixed, resulting in dry mass concentration comparable to the sponge, and heated in a microwave until boiling. Extract equivalent to 20 ml sponge tissue was dissolved in 2 ml MeOH and stirred into the artificial food mix after it had cooled down. The food mix was then poured into a mold. Control food was treated in the same way, but only 2 ml solvent was added to the artificial diet. The mold was 2 mm thick with two 2.5 x 25 cm openings cut into it. The openings in the mold were filled with treated or control food, according to the actual assay (see below). A piece of window screen (mesh size 1 mm) was clamped between the mold and wax paper. When the agar had cooled completely it was firmly attached to the screen. The screen was then cut perpendicular to the food strips, resulting in screens containing rectangles of one treated and one control food strip (2.5 x 2.0 cm). To determine the amount of control and treated food eaten, the squares in the window screen served as a grid, and the number of squares where the food had been completely removed was counted. Replicates with no visible feeding marks were disregarded (always ≤ 2). The food strips were checked periodically and pulled out when at least half of the total food mass was consumed, but not exceeding 3 hours. Following pairwise assays were conducted for each sponge species: Non-activated (representing the physiological state of an intact freshly collected specimen) vs. control food to test for constitutive feeding defense (except *Aplysinella* sp., where non-induced sponges were used for this assay), activated vs. non-activated to identify activated defenses and induced vs. non-induced to demonstrate increased deterrence due to defense induction. Two predators were used as bioassay organisms. The reef fish community was represented by the pufferfish *Canthigaster solandri*. *C*. *solandri* is very common around Guam, feeding on sponges, ascidians, other invertebrates and benthic algae (Schupp, pers. obs., [48]). This species represents a good model for feeding preferences of benthic reef fishes, since field feeding assays with the reef fish community and pufferfish assays provide congruent results [49]. For each feeding assay ten out of a total of 15 individuals of *C*. *solandri* (7.1–7.6 cm total length) were randomly chosen and kept individually in 70 L flow-through tanks and fed on the days prior to the feeding assays to avoid the loss of preference patterns [50]. The sponge *Aplysinella* sp. was not tested in the assay with the pufferfish *C*. *solandri*, because this assay had already been conducted by Thoms and Schupp [33]. Instead we reported the results from this study. Sea urchins (*Diadema savignyi*) were used to identify the defensive effects on benthic invertebrates. Feeding assays were performed in a similar way as the assays with *C*. *solandri*. A starvation period of one day was applied before every feeding assay to ensure feeding within three hours and all feeding assays were run at night, since the urchins started grazing mostly at night. The results of the feeding assays were analyzed using Wilcoxon’s signed rank test.

### Bacterial isolation, conservation and identification

Bacteria from the natural environment were isolated to identify differences in antimicrobial activity among sponge extracts. Bacterial strains were isolated from *Hydrolithon reinboldii*, a crustose coralline alga (CCA). Those CCA form an abundant reef substrate on which many sessile organisms settle. CCA were collected in Luminao, which is located outside of Apra Harbor, Guam, USA (13°27'53.57"N, 144°38'54.81"E). We inoculated different media swabs from the CCA shortly after the algae were collected. The media were incubated at 27°C and strains picked based on their morphology and color to establish a broad range of environmental bacterial isolates. Picked colonies were transferred at least three times to obtain pure isolates. In order to identify the isolates, DNA of each strain was extracted and DNA was purified using the QIAquick PCR Purification Kit (Qiagen, Gaithersburg, MD). PCR products were sequenced by Macrogen Inc. High-quality sequences (defined as >600 bp) were compared to the National Center for Biotechnology Information (NCBI) database (http://blast.ncbi.nlm.nih.gov) by using BLAST.

To preserve the isolates, cryo cultures were established for every pure strain. We chose 38 strains over a wide phylogenetic range and included all isolated strains with known pathogenic activities (mostly *Pseudoalteromonas* spp. and *Vibrio* spp.) in our assays. The selected strains were used in disc diffusion assays to identify bacterial growth inhibition of sponge extracts.

### Disc diffusion assays

For the disc diffusion tests sterile 6 mm discs for antibiotic tests (Chemie Roth) were used. These discs can hold a liquid volume of 30 μl. The sponge extracts were dissolved in methanol to obtain extracts in natural volumetric concentrations. 30μl of extract solution was applied to a disc. While the methanol evaporated, the media dishes were inoculated with 100 μl of 48h old liquid cultures of the test isolates. Pure Marine Broth media was used for the tests. After inoculation the dried discs were placed upside down onto the media. Each test was run in triplicates. 30 μl methanol was used as negative control. The antibiotic Rifampicine (10 mg/ml in methanol) was used as a positive control for gram positive strains. The positive control for gram negative strains was Gentamicine (1 mg/ml in sterile water). The plates were sealed with parafilm and incubated at 27°C. After 24 h and 48 h the plates were checked and inhibition zones were measured to the nearest 0.5 mm using a ruler. The means of the triplicates were used for analysis. The non-activated extracts represent extracts from untreated sponges and were used as a proxy for the main antimicrobial activity of the sponge species. To analyze whether the antimicrobial activity increased by activation or induction, pairwise comparisons were conducted between activated and non-activated, as well as between induced and non-induced extracts (Wilcoxon’s signed rank test).

## Results

### Feeding defenses

**Fig 1:**
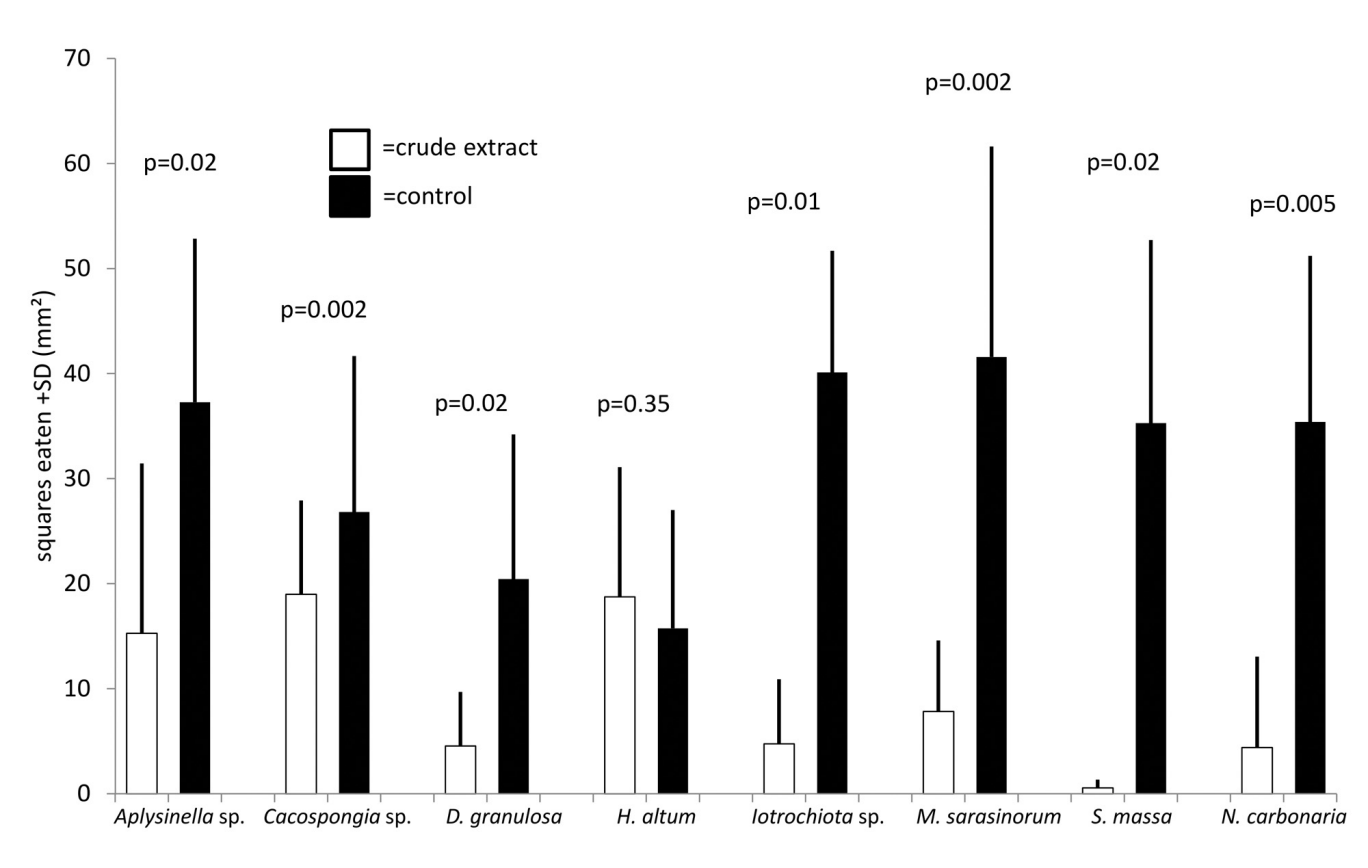
Effect of crude extract of sponges on predation by C*anthigaster solandri* (mean+1 SD, n = 10). Food pellets contained organic extracts at natural volumetric concentrations (white bars) or solvent only (black bars). P-values indicate the result of the pair wise analysis (Wilcoxon's signed rank test).

**Fig 2:**
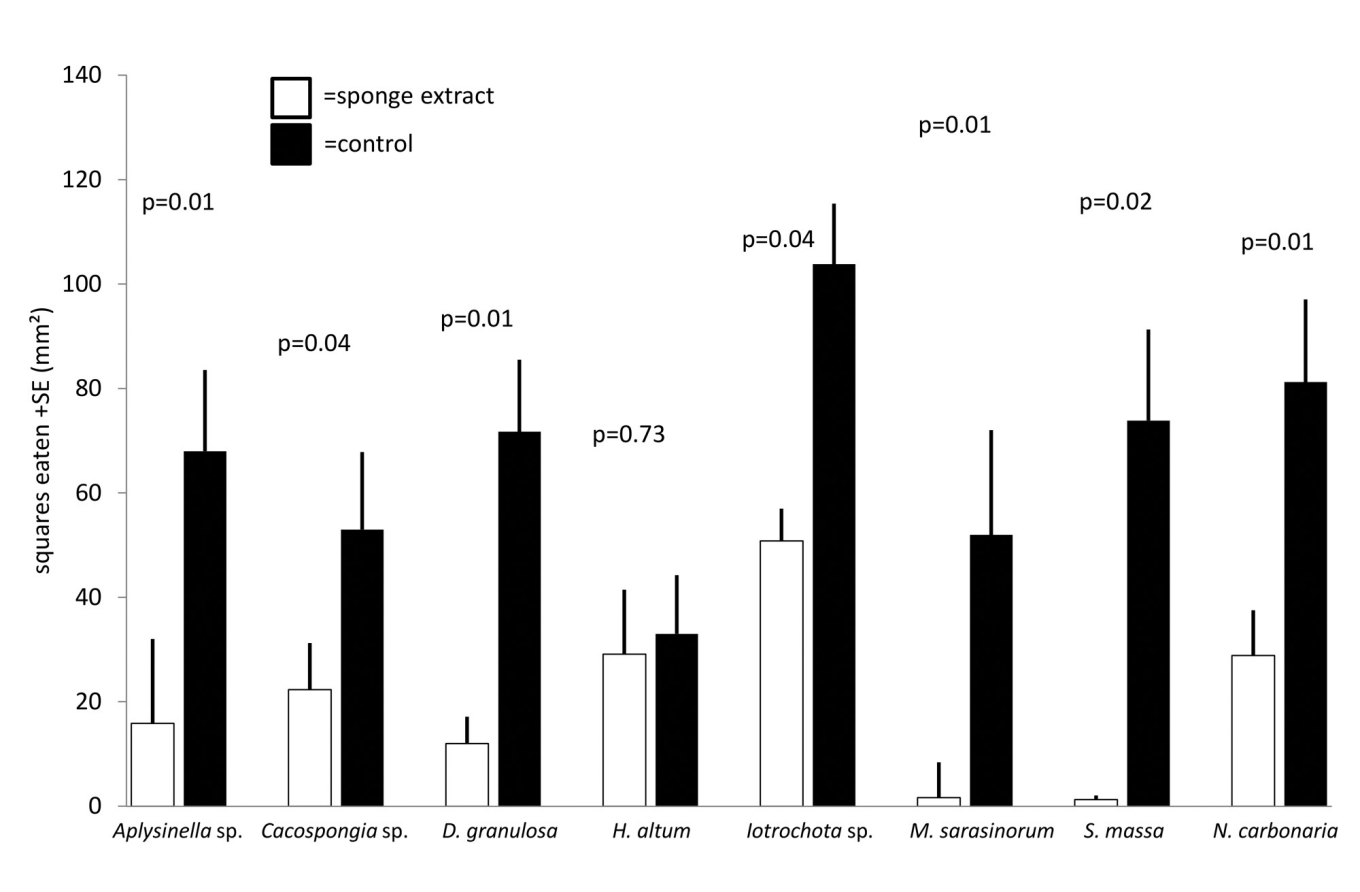
Effect of crude extract of sponges on predation by *Diadema savignyi* (mean+1 SD, n = 10). Food pellets contained organic extracts at natural volumetric concentrations (white bars) or solvent only (black bars). P-values indicate the result of the pair wise analysis (Wilcoxon's signed rank test).

General deterrence was tested by feeding assays with extracts of untreated sponges. All tested sponge species were chemically defended against predation compared to solvent controls (Wilcoxon, p<0.05), except for *H*. *altus*, that showed no antipredatory defenses (Wilcoxon, p = 0.35, Figs 1 and 2). The feeding assays with the pufferfish and the urchins produced highly congruent results.

**Fig 3:**
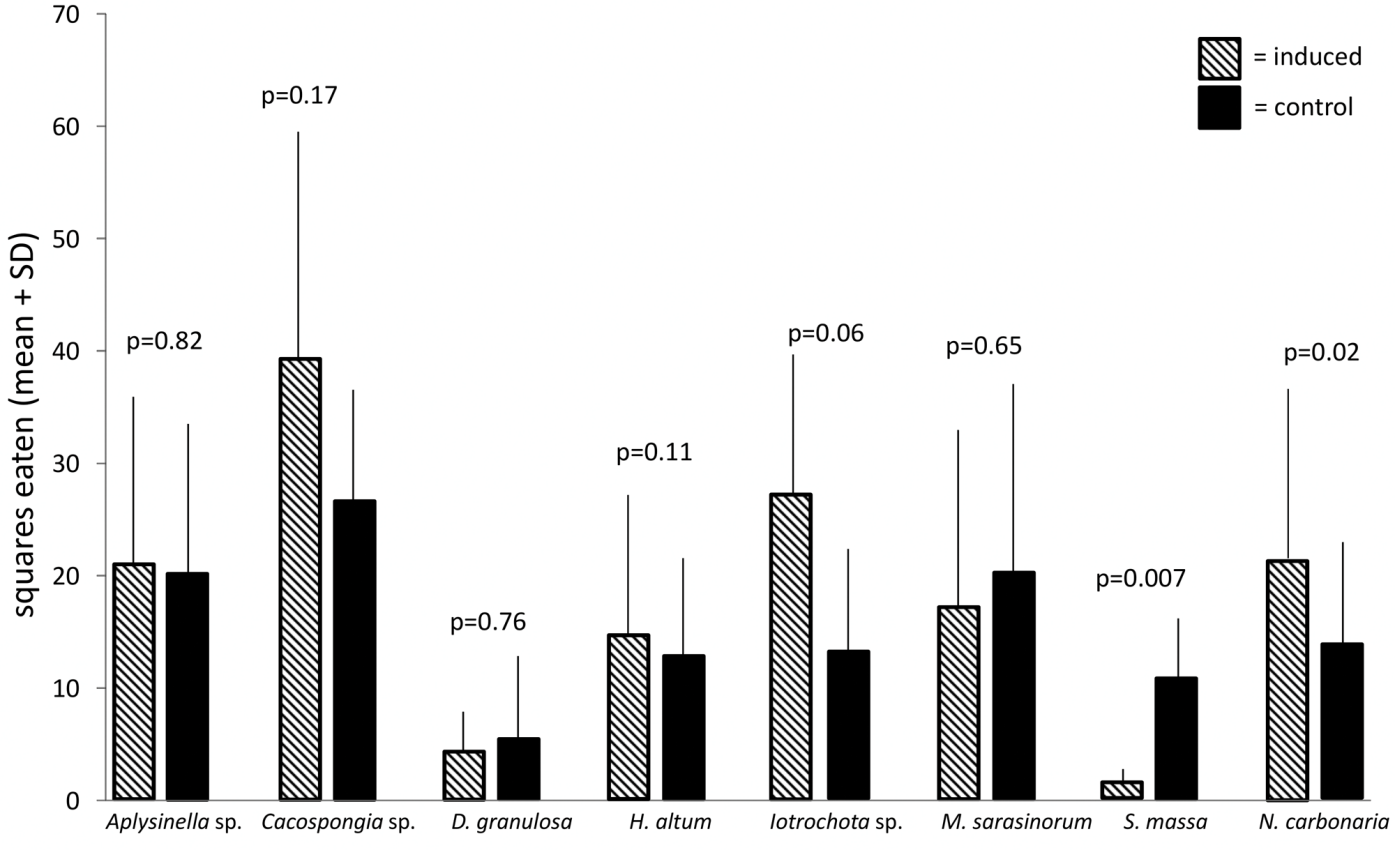
Effect of defense induction on predation by C*anthigaster solandri* (mean+1 SD, n = 10). Food pellets contained organic extracts of induced specimen (hatched bars) or non-induced specimen (black bars). P-values indicate the result of the pair wise analysis (Wilcoxon's signed rank test).

**Fig 4:**
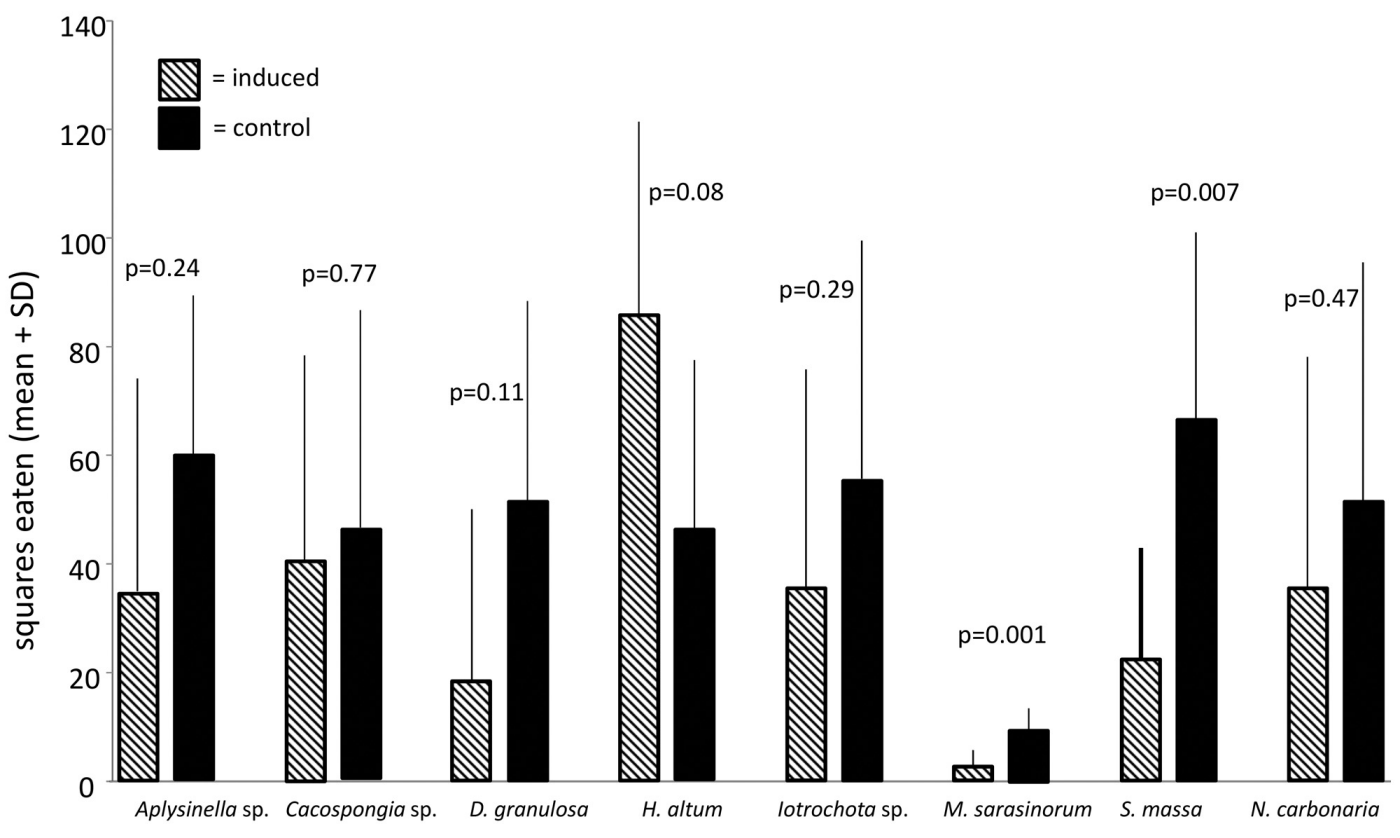
Effect of defense induction on predation by *Diadema savignyi* (mean+1 SD, n = 10). Food pellets contained organic extracts of induced specimen (hatched bars) or non-induced specimen (black bars). P-values indicate the result of the pair wise analysis (Wilcoxon's signed rank test).

Comparisons of the deterrence of induced sponges by simulated predation with non-induced sponges revealed that inducible defense was apparent only in the sponges *S*. *massa* and *M*. *sarasinorum* (Figs 3 and 4). While the induced defense of *S*. *massa* was effective on both the pufferfish and the urchins, the induced defense of *M*. *sarasinorum* was only effective in the pufferfish assays. The species *Aplysinella* sp., *Cacospongia* sp., *D*. *granulosa*, and *Iotrochota* sp. showed no difference in palatability in response to induction (Wilcoxon, p>0.05). *N*. *carbonaria* was even preferred by the pufferfish when it was induced compared to non-induced extracts (Wilcoxon, p = 0.02), but this effect was absent in the urchin assay.

**Fig 5:**
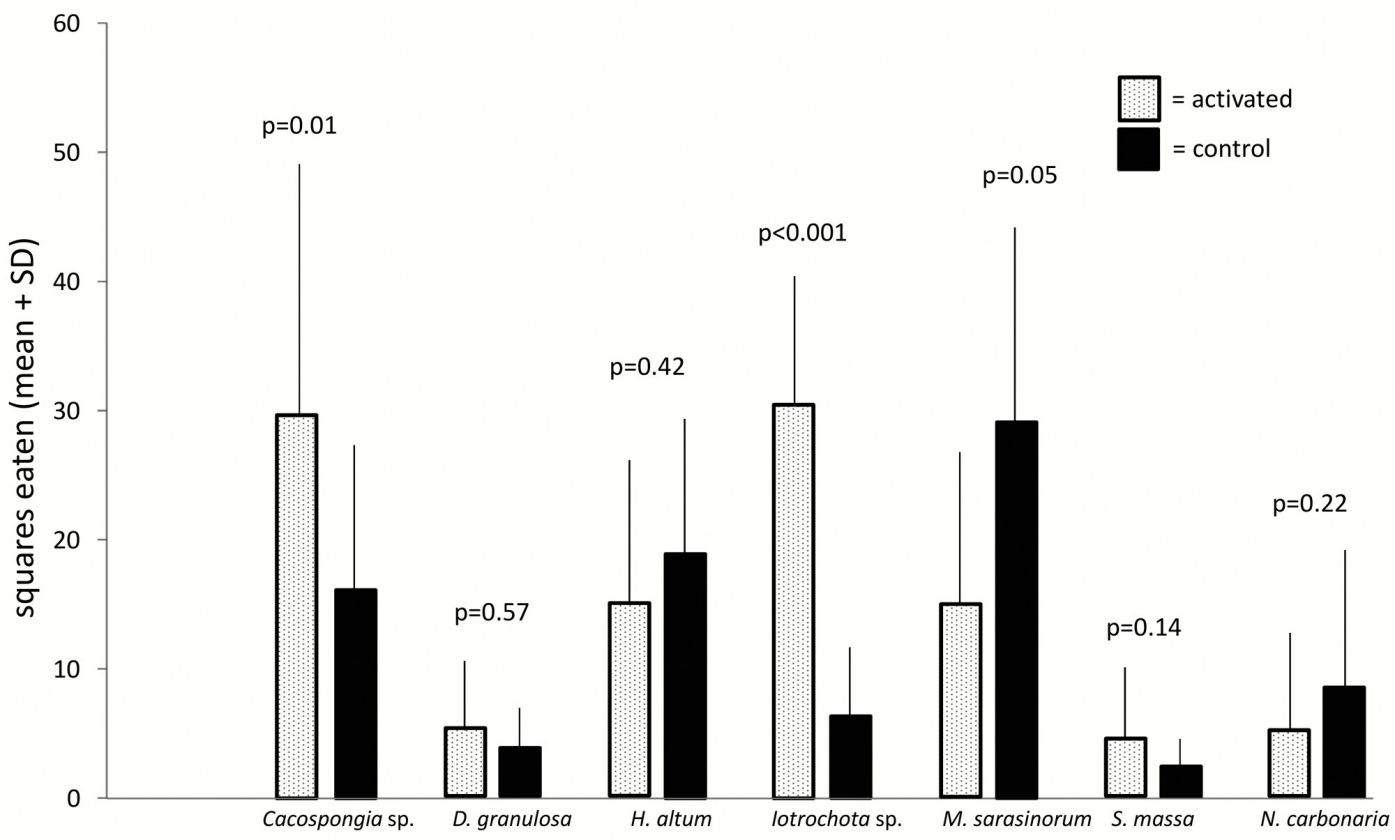
Effect of defense activation on predation by C*anthigaster solandri* (mean+1 SD, n = 10). Food pellets contained organic extracts of activated specimen (dotted bars) or intact specimen (black bars). P-values indicate the result of the pair wise analysis (Wilcoxon's signed rank test).

**Fig 6:**
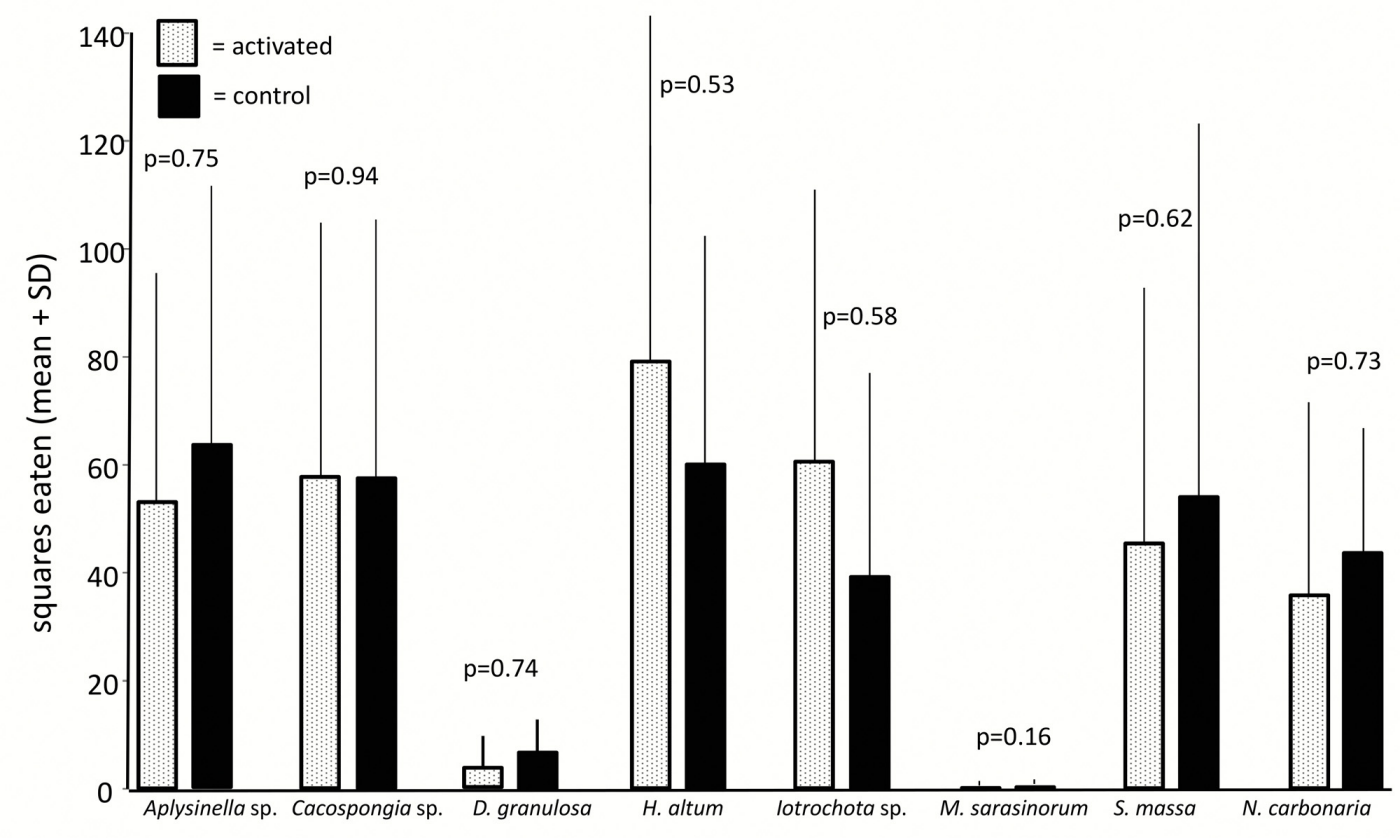
Effect of defense activation on predation by *Diadema savignyi* (mean+1 SD, n = 10). Food pellets contained organic extracts of activated specimen (dotted bars) or non-activated specimen (black bars). P-values indicate the result of the pair wise analysis (Wilcoxon's signed rank test).

Defense activation was indicated in the sponge *M*. *sarasinorum*. The pufferfish preferred non-activated extracts compared to activated ones (Fig 5; Wilcoxon, p = 0.05). This preference was not supported in the urchin assay, which could be due to the fact that the feeding rates of the urchins in this assay were very low (Fig 6). *Aplysinella* sp., *Cacospongia* sp., *D*. *granulosa*, *H*. *altus*, *Iotrochota* sp., *S*. *massa*, and *N*. *carbonaria* revealed no activated defense mechanisms. Activated extracts of *Cacospongia* sp. and *Iotrochota* sp. were even preferred by the pufferfish (Wilcoxon, p<0.01), but not by the urchins.

### Antimicrobial activity

**Fig 7:**
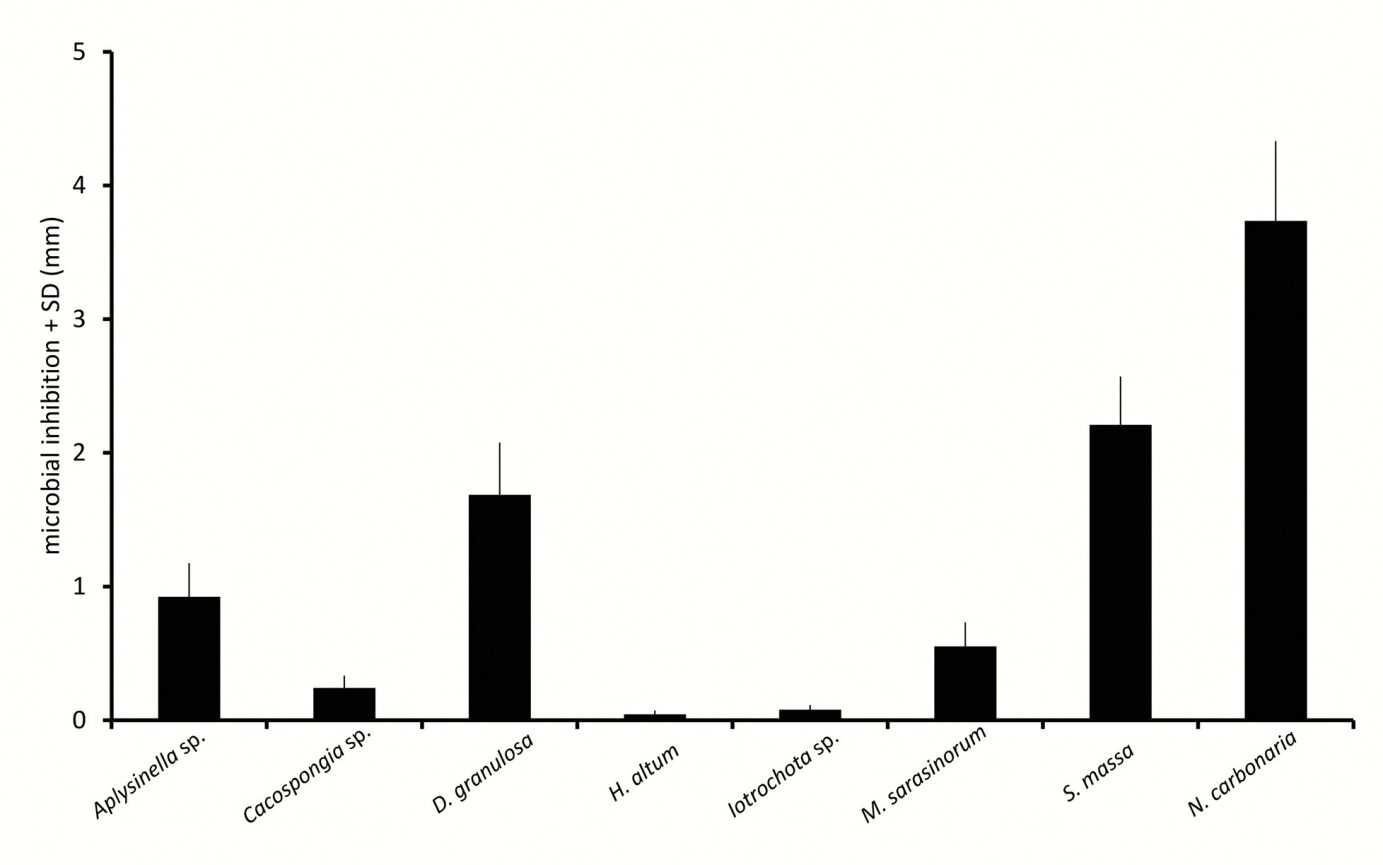
Microbial inhibition of sponge extracts in disc diffusion assays.

**Fig 8:**
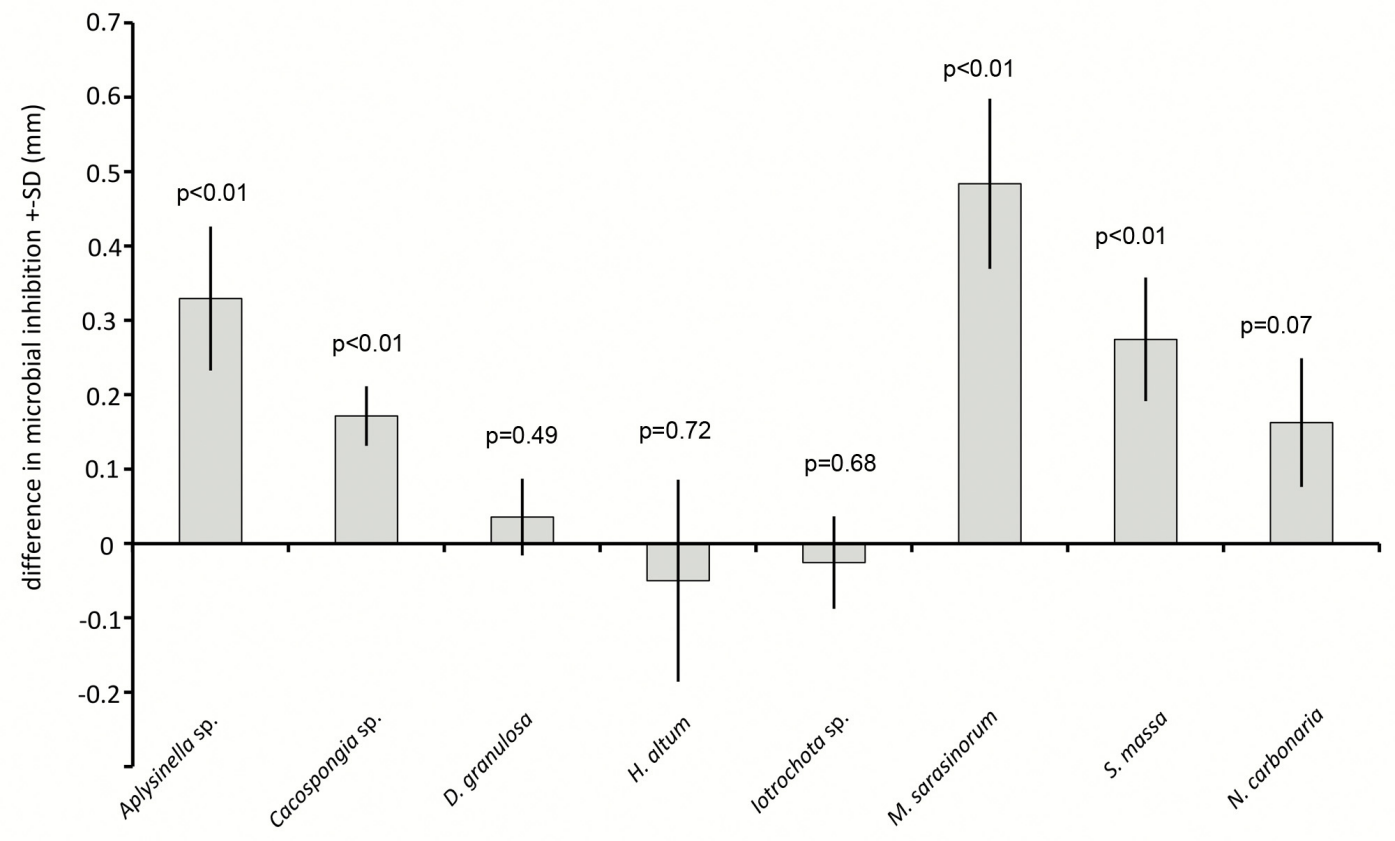
Difference in microbial inhibition between induced and non-induced sponge extracts in disc diffusion assays. Positive values reflect higher inhibition in induced extracts, negative values reflect higher inhibition in non-induced extracts. P-values indicate the result of the pair wise analysis (Wilcoxon's signed rank test).

**Fig 9:**
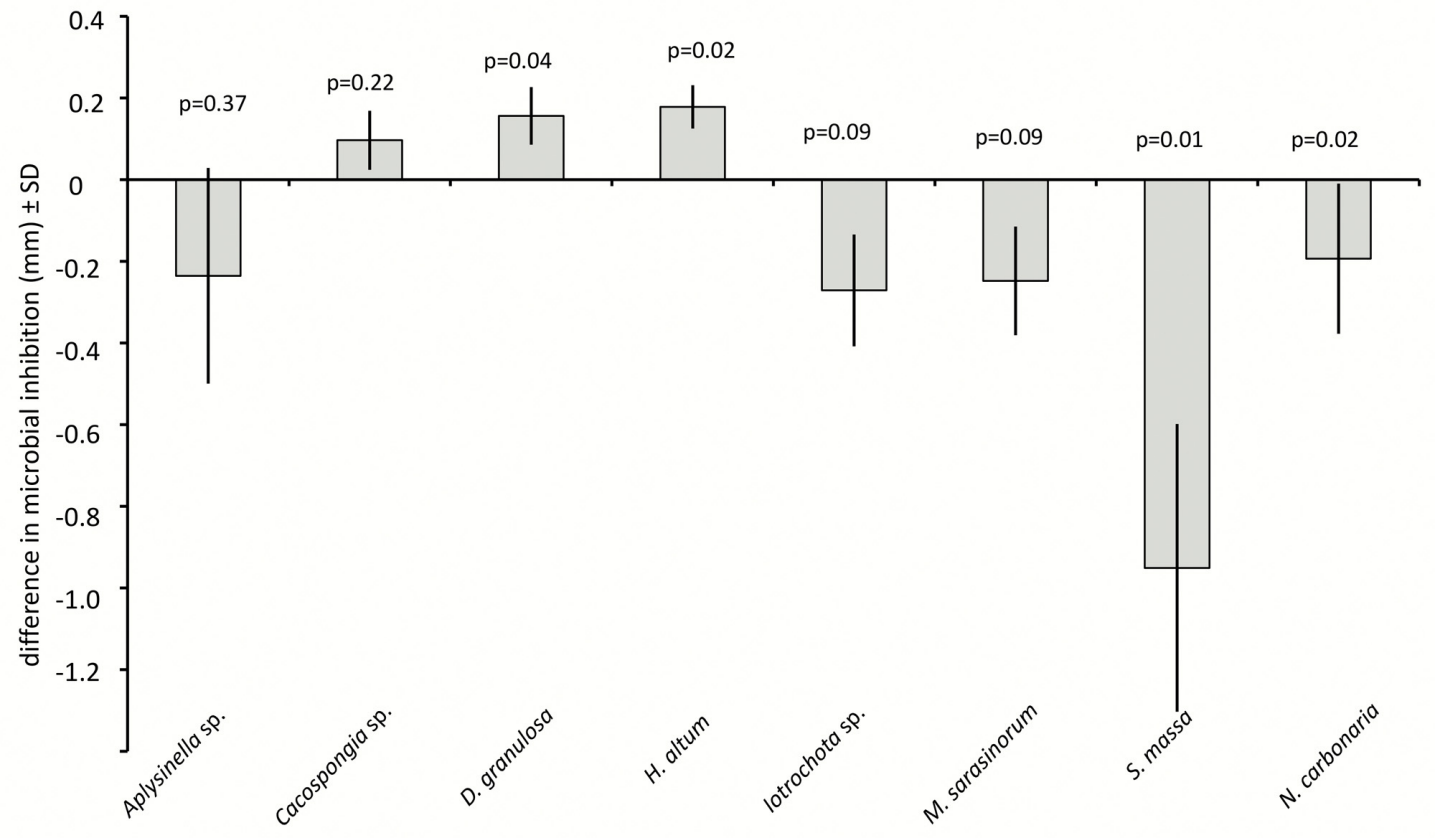
Difference in microbial inhibition between activated and non-activated sponge extracts in disc diffusion assays. Positive values reflect higher inhibition in induced extracts; negative values reflect higher inhibition in non-induced extracts. P-values indicate the result of the pair wise analysis (Wilcoxon's signed rank test).

Disc diffusion assays investigated the antimicrobial activity of sponge extracts (Fig 7). Comparisons between sponge species showed very variable antimicrobial effects. *H*. *altus* and *Iotrochota* sp. showed very low antimicrobial activity, *M*. *sarasinorum* and *Aplysinella* sp. medium and *D*. *granulosa*, *S*. *massa* and *N*. *carbonaria* rather strong antimicrobial activity. Induction treatment increased microbial inhibition significantly in four of the eight tested sponge species (Fig 8), with *Aplysinella* sp., *Cacospongia* sp., *M*. *sarasinorum*, and *S*. *massa* yielding higher antimicrobial activity (Wilcoxon, p<0.01). *N*. *carbonaria* showed a similar trend (Wilcoxon, p = 0.07), while *D*. *granulosa*, *H*. *altus* and *Iotrochota* sp. showed no difference between induced and non-induced state. Activation treatment increased microbial inhibition in *D*. *granulosa* and *H*. *altus* significantly (Wilcoxon, p = 0.04, p = 0.02, resp.), decreased antimicrobial activity in *S*. *massa* and *N*. *carbonaria* (Wilcoxon, p = 0.01, p = 0.02, resp.) and did not change in *Aplysinella* sp., *Cacospongia* sp., *Iotrochota* sp., and *M*. *sarasinorum* (Fig 9, Wilcoxon, p>0.05).

## Discussion

Until the 1990s, marine ecologists commonly assumed that predation had little effects on sponge populations, since very few predators had been observed to feed on sponges [51, 52]. Later on, studies conducted in the Caribbean established that low predation rates were mainly a result of sponge chemical defenses [14], a fact that was also supported by other studies from the Pacific, Mediterranean, Red Sea and Antarctica [53–55]. In our study, 87% of the tested species were chemically defended, which is comparable to the results of the studies cited above and confirms the importance of chemical defenses to persist in predator rich environments such as tropical coral reefs.

While chemical deterrence in marine invertebrates and especially in sponges is a widely documented phenomenon, more specific defense mechanisms and defense dynamics have been seldom demonstrated in sponge-predator interactions. Many terrestrial and marine plants optimize their herbivore resistance by inducible or activated defense [56, 57]. We investigated the occurrence of these mechanisms in sponges, a group of sessile marine invertebrates, which share many ecological traits with plants. Similar to plants, they lack behavioral defenses and are often non-fatally attacked by predators [58]. One sponge species, *S*. *massa*, showed induced defense against feeding by both pufferfish and sea urchins. Additionally, induction of defense in *M*. *sarasinorum* reduced palatability in sea urchin assays. An induced increase of concentrations of deterrent compounds in response to wounding has been reported before in the sponge *Agelas conifera* [37]. The prevalence of inducible defense in the tested sponge species seems to be relatively low compared to the prevalence in marine macroalgae [18, 19]. *M*. *sarasinorum* was also the only sponge that showed an indication of activated defense with regard to urchin feeding. Defense activation has often been observed in terrestrial plants [56, 57] but only to a limited extent in marine algae and invertebrates [27, 31, 33, 59]. Therefore, we cannot reliably compare the prevalence rates of activated defense in macroalgae and sessile invertebrates. However, since most of the few existing examples of activated defenses in marine invertebrates were reported from sponges and we identified another case of activated defenses in sponges in this study, it seems that the occurrence of activated defenses in sponges is not an isolated phenomenon.

The sponge *S*. *massa* induced defense effectively against fish and urchin feeding. *S*. *massa* belongs to the family Dictyonellidae, which is a well investigated group of marine sponges from the perspective of natural products chemistry, and some ecological roles for identified compounds have even been identified [53]. The most prominent natural products isolated from *S*. *massa* belong to the group of alkaloids [45, 60–63]. It has been shown that alkaloids in *S*. *massa* vary at small and large scale, i.e. between sites and regions and that abiotic and biotic factors can affect alkaloid concentrations in this sponge [45]. The major compounds, oroidin, debromohymenialdisine, hymenialdisine, sceptrin, hymenidin, and palau’amine extracted from *S*. *massa* did not reveal antifeeding properties [45], even though some of them proved to be effective in earlier studies when extracted from other sponge species and tested with other bioassay organisms [64–67]. The high variabiltiy in compound concentration in *S*. *massa* [45] could partly explain the diverging results and induced defense may has been a factor which contributed to the variation.

*M*. *sarasinorum* was the other sponge that demonstrated induced defense. Numerous secondary metabolites have previously been isolated from *M*. *sarasinorum* for pharmacological research (e.g. [68, 69]), but nothing is known about the compound’s ecological functions. However, *M*. *sarasinorum* is chemically defended, since its crude extract deterred feeding of pufferfish and a natural reef fish community [49]. The deterrent effect was also observed in this study, and furthermore, simulated predation increased the deterrent effect in the urchin assay. It is known that defensive compounds may not be active against all predator species, which could explain the species-specific predator response to induced defenses in this study [25, 70]. Apparently, the sea urchins responded with more sensitivity than the pufferfish to the induced changes in *M*. *sarasinorum*’s chemistry.

Defense induction is a cost effective defense adaptation, since metabolically costly defensive metabolites are produced on demand only. However, the evolution of inducible defenses against predation requires special ecological conditions (reviewed in [20, 71, 72]). Predation pressure must be variable since constant pressure would select for a constitutive defense. Reducing defense should enhance fitness in the absence of consumers. And, finally, a reliable cue for triggering induction must exist. The tropics are characterized by a relative constant predation pressure compared to temperate regions, where seasonality provides an additional source for variability in predation pressure [20]. Consequently, one would assume little prevalence of inducible defenses in the tropics, which is supported by this study. Becerro et al. [53] conducted experiments in both tropical and temperate habitats to evaluate the deterrent properties of sponges. They found no experimental evidence that chemical defenses of tropical sponges are more deterrent than those of temperate sponges, suggesting that chemical defenses of temperate and tropical sponges are equally effective. However, induced defenses were not investigated in their study. Induction of defense may take days to weeks, which is a disadvantage compared to constitutive defense. During the inducing period the organism is relatively undefended. In order to make defenses effective, biomass loss during this period must be tolerated and should not be lethal. As a consequence, Hay [26] proposed that defense induction in benthic prey should be more common against smaller, less mobile mesograzers responsible for partial biomass loss. Predation on sponges has been reported to happen by larger predators like trunk fishes, angel fishes, or parrotfishes [51, 52, 73]. These species consume large biomasses in relatively short periods of time and are capable of rapidly consuming the prey, thus making a slow induction of defense futile. Both, the lack of pronounced seasonal variability in the tropics and the dominance of macropredators could explain the low number of induced defenses in this study. It would be interesting to conduct a similar study in temperate regions, where predator densities are more variable and the dominant predator groups consist of mesopredators like sea urchins, crustaceans and gastropods.

Reducing investments in defense mechanisms (e.g. secondary metabolite production) should increase metabolic resources for other processes like reproduction. Reproductive parameters were not analyzed in this study. However, sponges that are highly defended against predation appear to have a lower reproductive output compared to less defended species [17], indicating the costs of defenses and the potential advantage of inducible defenses.

Nearly all sponge species tested were feeding deterrent (except *H*. *altus*), preventing fish feeding and therefore natural induction by spongivorous fishes in aquarium experiments. As a consequence, clipping was used as defense inducing treatment. Several studies on macroalgae used clipping as a method to simulate herbivory (e.g. [25, 70, 74]). However, earlier reviews have already cautioned that clipping does not simulate herbivory adequately and it should not be assumed to do so without proper testing [75, 76]. Prey organisms may rely on specific chemical cues to ensure that defenses are only induced when predation risk is high [43]. It is possible that the simulated predation in our study lacked specific chemical cues, therefore not triggering defense induction in the tested species.

*M*. *sarasinorum* was the only species that revealed activated antipredatory defense mechanisms. The effect was only detectable in the pufferfish assay; the urchins did not distinguish between activated and non-activated extracts. One reason that an activated defense mechanism was not detected in the urchin assays can be the low feeding rates of the urchins, which could have masked any differences in this particular assay. Activated defense was not tested in *Aplysinella* sp. since it had been described in this species in detail before [33].

Defense activation is defined as the wound-activated conversion of inactive or less active secondary metabolites to more active forms with defensive functions [23]. Prerequisites are that the conversion is enzyme-catalyzed and that the precursor compound is stored either in different cells or different cell compartments than the catalyzing enzyme as to prevent continuous conversion of the precursors. Defense activation is a rather mechanical process, in which cell disruption triggers the biochemical reaction. No external chemical cues are required in contrast to induced defense. Consequently, high intensities of wounding, like grinding used in this study—even if ecologically irrelevant—provide a cytological worst-case scenario and can help to initially observe wound-activated chemical reactions [58]. If the cytological and chemical prerequisites for activated defense against predation are present, they should therefore have been detected in our assays. The ecological relevance of these indications would need further investigations. The three cases of activated defense (*Aplysina aerophoba*, *Aplysinella* sp., *M*. *saranisorum*), which have been reported in the literature and in this study [31, 33]indicate that the number of tropical sponge species that increase their constitutive defense by activated defense is low.

While cost saving is a clear advantage for induced defense systems, the advantages of activated defenses are less obvious. In activated defense systems, biologically active compounds are stored in different compartments. During wounding and disruption of compartmentalization by predators, the biologically less active compounds are converted to potent compounds. Therefore the organisms can avoid or reduce autotoxicity, as they only store less active compounds for bioconversion [59]. The high number of constitutive defenses in this and other studies suggests that sponges deal well with potential autotoxic effects of defensive compounds. Further studies on the autotoxic effects of precursor and converted defensive compounds could reveal whether constitutive defenses are more common due to ecological or physiological reasons.

Sponges are known to produce more than 5000 secondary metabolites [77] and it is recognized that many species have elaborate chemical defenses to deter possible predators and also to inhibit the colonization, growth, or pathogenicity of harmful microbes [78]. In the present study, 75% of all tested sponge species demonstrated antimicrobial activity.

In various cases, facultative antimicrobial defenses have been reported. In the sponge *Suberites domuncula*, compounds with strong antimicrobial and antifungal activity increased after exposure to endotoxins derived from gram-negative bacteria [79]. Induced antimicrobial defense was found in the sponge *Agelas conifera*, where wounding induced an increase of the antimicrobial compounds sceptrin and oroidin [37]. Sceptrin and oroidin have also been identified in *S*. *massa* [45]. The concentrations of these compounds were not analyzed in this study, but wounding induced an increased antimicrobial activity in *S*. *massa* suggesting a similar reaction. Activated antimicrobial defense has been reported from the sponge *Aplysina aerophoba*. The bioconversion of isoxazoline alkaloids following cell disruption lead to an increase in antibiotic and cytotoxic activity [32, 80]. *A*. *aerophoba* is a closely related species to *Aplysinella* sp., which has been shown to harbor an activated antipredatory defense by converting psammaplin A sulfate to psammaplin A [33]. Psammaplins, in particular psammaplin A have demonstrated cytotoxic properties. Interestingly, activation did not increase antimicrobial activity in our study, suggesting that psammaplin A sulfate and psammaplin A possess similar antimicrobial properties. The activation of antipathogen defense would be especially effective, since the bioconversion products are formed only in injured cells, where a penetration by pathogenic microorganisms is most likely to occur. *D*. *granulosa* and *H*. *altus* showed significantly higher antimicrobial activity in activated extracts, supporting this theory. The activity of four tested sponge species did not differ between activated and non-activated extracts, and two species even had higher antimicrobial activity in the non-activated treatments. One reason for this variable result may be that antibacterial compounds in sponges are highly selective. This selectivity may serve to establish natural sponge-microbial associations, while inhibiting settlement or growth of potential pathogens [45]. The lack of known sponge pathogenic bacteria necessitated the use of sympatric environmental strains in this study. The use of specific sponge pathogens in future studies may reveal a different pattern.

Interestingly, 50% of the tested sponge species demonstrated induced antimicrobial defense. Simulated predation increased the antimicrobial activity of *Aplysinella* sp., *Cacospongia* sp., *M*. *sarasinorum*, and *S*. *massa*. With increased wounding intensity, the risk of pathogen infection via feeding scars rises, while it can also lead to increased antimicrobial defenses through induced or activated defense mechanisms. These results can have consequences for future ecological and pharmaceutical studies on antimicrobial compounds from sponges. We demonstrated that wounding frequently can alter antimicrobial chemistry in sponges, which needs to be addressed in the handling of sponges and the design of ecological experiments. Pharmaceutical studies that are interested in the extraction and isolation of bioactive compounds could increase amounts of antimicrobial compounds by inducing compound production prior to collection and extraction.

In conclusion, we could demonstrate that both induced and activated antipredatory defenses are present in tropical sponges. However, constitutive defense appears to outweigh induced or activated mechanisms in tropical sponges. Future studies should reveal if ecological factors like predator community composition or variability in predation intensity are the driving force in that pattern, or whether physiological constraints limit facultative defenses in sponges. Furthermore, we could show that the production of antimicrobial compounds was also increased by induced and activated defenses in many sponge species. To identify the underlying signaling pathways for these responses could be a rewarding task for future studies and give more insights in the immune systems of marine invertebrates.

## Supporting Information

S1 Table Results of the feeding assays. (XLSX)

S2 Table Disc diffusion assays. (XLSX)
